# 2-(4-Bromo­phen­yl)-5-dodec­yloxy-1,3-thia­zole

**DOI:** 10.1107/S1600536810032666

**Published:** 2010-08-21

**Authors:** Hugo Gallardo, Deise M. P. O. Santos, Adailton J. Bortoluzzi

**Affiliations:** a Departamento de Química, Universidade Federal de Santa Catarina, 88040-900 Florianópolis, SC, Brazil

## Abstract

In the structure of the title compound, C_21_H_30_BrNOS, an important inter­mediate for the preparation of liquid crystal compounds, the saturated C_12_ chain shows a linear conformation while the benzene and thia­zole rings are essentially coplanar [dihedral angle = 4.5 (4)°]. The crystal packing shows no significant inter­molecular inter­actions.

## Related literature

For technological applications of liquid crystals, see: Sonar *et al.* (2008 ▶); Srivastava *et al.* (2008 ▶). For liquid-crystalline compounds containing heterocyclic units, see: Cristiano *et al.* (2006 ▶); Kauhanka & Kauhanka (2006 ▶); Vieira *et al.* (2008 ▶). For the properties of thia­zole derivatives, see: Gallardo *et al.* (2008 ▶); Yamashita (2010 ▶); Parra *et al.* (2001 ▶); Cohen *et al.* (2010 ▶). For the synthesis, see: Kiryanov *et al.* (2001 ▶). For related structures, see: Metzger (1984 ▶); Krapivin *et al.* (1992 ▶).
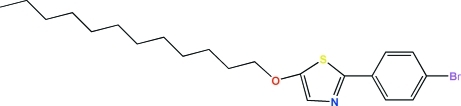


## Experimental

### 

#### Crystal data

C_21_H_30_BrNOS
*M* *_r_* = 424.43
Monoclinic, 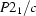

*a* = 5.507 (1) Å
*b* = 46.999 (6) Å
*c* = 8.326 (1) Å
β = 99.68 (1)°
*V* = 2124.3 (5) Å^3^
*Z* = 4
Mo *K*α radiation
μ = 2.04 mm^−1^
*T* = 293 K
0.47 × 0.47 × 0.36 mm

#### Data collection

Enraf–Nonius CAD-4 diffractometer
Absorption correction: ψ scan [North *et al.* (1968 ▶) and *PLATON* (Spek, 2009 ▶)] *T*_min_ = 0.447, *T*_max_ = 0.527
4001 measured reflections
3740 independent reflections
1486 reflections with *I* > 2σ(*I*)
*R*_int_ = 0.101
3 standard reflections every 25 reflections  intensity decay: 1%

#### Refinement

*R*[*F* ^2^ > 2σ(*F* ^2^)] = 0.081
*wR*(*F* ^2^) = 0.248
*S* = 1.05
3740 reflections
227 parameters
H-atom parameters constrained
Δρ_max_ = 0.61 e Å^−3^
Δρ_min_ = −0.47 e Å^−3^

### 

Data collection: *CAD-4 Software* (Enraf–Nonius, 1989 ▶); cell refinement: *SET4* in *CAD-4 Software*; data reduction: *HELENA* (Spek, 1996 ▶); program(s) used to solve structure: *SIR97* (Altomare *et al.*, 1999 ▶); program(s) used to refine structure: *SHELXL97* (Sheldrick, 2008 ▶); molecular graphics: *PLATON* (Spek, 2009 ▶); software used to prepare material for publication: *SHELXL97*.

## Supplementary Material

Crystal structure: contains datablocks global, I. DOI: 10.1107/S1600536810032666/zs2055sup1.cif

Structure factors: contains datablocks I. DOI: 10.1107/S1600536810032666/zs2055Isup2.hkl

Additional supplementary materials:  crystallographic information; 3D view; checkCIF report

## Figures and Tables

**Fig. 1.**
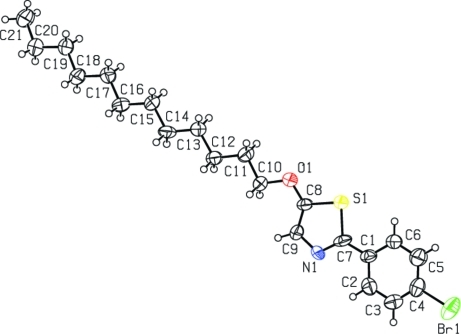
The molecular structure of the title compound with atom labelling scheme. Displacement ellipsoids are shown at the 40% probability level.

## supplementary crystallographic information

### Comment

Liquid crystals are fascinating materials with a broad range of applications. The most famous application of these materials is in liquid crystal displays (LCDs), but over the past year new applications have appeared, such as organic light emitting diodes (OLEDs) (Sonar *et al.*, 2008; Srivastava *et al.*, 2008). The molecular shape has a dominant influence on the existence of the liquid crystalline state. Over several years a large number of liquid-crystalline compounds containing heterocyclic units have been synthesized (Cristiano *et al.*, 2006). Heterocycles are of great importance as core units in thermotropic liquid crystals due to their ability to impart lateral and/or longitudinal dipoles combined with changes in the molecular shape. The incorporation of heteroatoms can also result in large changes in the corresponding liquid crystalline phases and/or in the physical properties of the observed phases, because most of the heteroatoms (S, O, and N) commonly introduced are chemically classified as more polarizable than carbon (Kauhanka & Kauhanka, 2006; Vieira *et al.*, 2008). As part of our studies of liquid crystal derivatives of thiazoles, we now report the synthesis and structure of the title compound C_21_H_30_BrNOS (I). In (I) (Fig. 1), the saturated C_12_ chain shows a linear conformation while the benzene and thiazole rings are essentially coplanar [dihedral angle, 4.5 (4)°]. The crystal packing shows no significant intermolecular interactions.

### Experimental

Dodecyl-2-(4-bromobenzamido)acetate (4.26 g, 10 mmol) and 2,4-bis(4-methoxyphenyl)-1,3,2,4-dithiadiphosphetane-2,4-disulfide (Lawesson's reagent) (8.10 g, 20 mmol) was mixed with dry toluene (150 ml) and heated under reflux for 6 h. The crude product was separated by column chromatography (dichloromethane) and after evaporation of the solvent the solid was recrystallized from methanol to afford the title compound as a white solid (3.8 g, 95%): ^1^H NMR (CDCl_3_) = 7.64 (d, J = 8.0 Hz, 2H), 7.48 (d, J = 8.0 Hz, 2H), 7.06 (s, 1H), 4.03 (t, J = 6.5 Hz, 2H), 1.81 (q, J = 7.0 Hz, 2H), 1.52–1.20 (m, 18H), 0.89 (t, J = 6.8 Hz, 3H); Elemental analysis for C_21_H_30_BrNOS: calc.: C 59.43; H 7.12; N 3.30; S 7.55%. Found: C 59.42; H 7.16; N 3.43; S 8.25%.

### Refinement

All non-H atoms were refined with anisotropic displacement parameters. H atoms were placed at their idealized positions with C—H_Ar_ = 0.93 Å, C—H_methylene_ = 0.97 Å and C—H_methyl_ = 0.96 Å and treated as riding, with *U*_iso_ = 1.2 or 1.5 times *U*_eq_(C) for aromatic/methylene and methyl groups, respectively.

### Crystal data

**Table:**

C_21_H_30_BrNOS	*F*(000) = 888
*M**_r_* = 424.43	*D*_x_ = 1.327 Mg m^−^^3^
Monoclinic, *P*2_1_/*c*	Mo *K*α radiation, λ = 0.71069 Å
Hall symbol: -P 2ybc	Cell parameters from 25 reflections
*a* = 5.507 (1) Å	θ = 3.8–11.5°
*b* = 46.999 (6) Å	µ = 2.04 mm^−^^1^
*c* = 8.326 (1) Å	*T* = 293 K
β = 99.68 (1)°	Block, colorless
*V* = 2124.3 (5) Å^3^	0.47 × 0.47 × 0.36 mm
*Z* = 4	

### Data collection

**Table:**

Enraf–Nonius CAD-4 diffractometer	1486 reflections with *I* > 2σ(*I*)
Radiation source: fine-focus sealed tube	*R*_int_ = 0.101
graphite	θ_max_ = 25.0°, θ_min_ = 0.9°
ω–2θ scans	*h* = −6→6
Absorption correction: ψ scan [North *et al.* (1968) and *PLATON* (Spek, 2009)]	*k* = −55→0
*T*_min_ = 0.447, *T*_max_ = 0.527	*l* = −9→0
4001 measured reflections	3 standard reflections every 25 reflections
3740 independent reflections	intensity decay: 1%

### Refinement

**Table:**

Refinement on *F*^2^	Primary atom site location: structure-invariant direct methods
Least-squares matrix: full	Secondary atom site location: difference Fourier map
*R*[*F*^2^ > 2σ(*F*^2^)] = 0.081	Hydrogen site location: inferred from neighbouring sites
*wR*(*F*^2^) = 0.248	H-atom parameters constrained
*S* = 1.05	*w* = 1/[σ^2^(*F*_o_^2^) + (0.1139*P*)^2^] where *P* = (*F*_o_^2^ + 2*F*_c_^2^)/3
3740 reflections	(Δ/σ)_max_ < 0.001
227 parameters	Δρ_max_ = 0.61 e Å^−^^3^
0 restraints	Δρ_min_ = −0.47 e Å^−^^3^

### Fractional atomic coordinates and isotropic or equivalent isotropic displacement parameters (Å^2^)

**Table:**

	*x*	*y*	*z*	*U*_iso_*/*U*_eq_	
C1	−0.4151 (15)	0.5923 (2)	−0.0151 (9)	0.045 (2)	
C2	−0.6447 (17)	0.5928 (2)	−0.1107 (10)	0.055 (2)	
H2	−0.7290	0.5758	−0.1357	0.067*	
C3	−0.7521 (17)	0.6180 (2)	−0.1703 (11)	0.064 (3)	
H3	−0.9067	0.6177	−0.2356	0.077*	
C4	−0.6371 (19)	0.6430 (2)	−0.1357 (11)	0.059 (3)	
C5	−0.4059 (19)	0.6436 (2)	−0.0409 (12)	0.067 (3)	
H5	−0.3244	0.6609	−0.0184	0.081*	
C6	−0.2960 (16)	0.6186 (2)	0.0206 (10)	0.057 (2)	
H6	−0.1417	0.6191	0.0862	0.068*	
C7	−0.3088 (14)	0.5663 (2)	0.0520 (9)	0.050 (2)	
C8	−0.0587 (16)	0.52788 (19)	0.1924 (10)	0.051 (2)	
C9	−0.2786 (16)	0.51987 (19)	0.1131 (11)	0.057 (3)	
H9	−0.3338	0.5012	0.1129	0.069*	
C10	0.0700 (15)	0.48362 (19)	0.2965 (10)	0.053 (2)	
H10A	0.0349	0.4753	0.1885	0.063*	
H10B	−0.0741	0.4812	0.3480	0.063*	
C11	0.2887 (16)	0.46862 (19)	0.3963 (11)	0.056 (2)	
H11A	0.4332	0.4714	0.3456	0.067*	
H11B	0.3222	0.4768	0.5046	0.067*	
C12	0.2390 (14)	0.43752 (19)	0.4087 (10)	0.051 (2)	
H12A	0.2078	0.4295	0.2999	0.061*	
H12B	0.0913	0.4350	0.4562	0.061*	
C13	0.4524 (15)	0.42099 (19)	0.5121 (11)	0.054 (2)	
H13A	0.6000	0.4237	0.4647	0.065*	
H13B	0.4828	0.4291	0.6207	0.065*	
C14	0.4093 (14)	0.3900 (2)	0.5259 (11)	0.060 (3)	
H14A	0.3704	0.3821	0.4172	0.072*	
H14B	0.2670	0.3873	0.5788	0.072*	
C15	0.6280 (16)	0.37363 (19)	0.6215 (11)	0.057 (2)	
H15A	0.7714	0.3774	0.5713	0.069*	
H15B	0.6618	0.3813	0.7311	0.069*	
C16	0.6002 (16)	0.3421 (2)	0.6340 (11)	0.059 (3)	
H16A	0.5517	0.3343	0.5253	0.071*	
H16B	0.4686	0.3381	0.6949	0.071*	
C17	0.8306 (16)	0.32698 (18)	0.7151 (12)	0.063 (3)	
H17A	0.9627	0.3315	0.6555	0.075*	
H17B	0.8767	0.3346	0.8244	0.075*	
C18	0.8118 (16)	0.2954 (2)	0.7266 (11)	0.068 (3)	
H18A	0.7613	0.2877	0.6180	0.082*	
H18B	0.6848	0.2908	0.7901	0.082*	
C19	1.0484 (16)	0.28141 (19)	0.8031 (11)	0.066 (3)	
H19A	1.0943	0.2886	0.9133	0.079*	
H19B	1.1768	0.2870	0.7427	0.079*	
C20	1.0392 (17)	0.2490 (2)	0.8096 (13)	0.071 (3)	
H20A	0.9186	0.2435	0.8761	0.086*	
H20B	0.9829	0.2419	0.7003	0.086*	
C21	1.2787 (18)	0.2351 (2)	0.8758 (17)	0.104 (4)	
H21A	1.3474	0.2438	0.9777	0.156*	
H21B	1.3906	0.2374	0.7999	0.156*	
H21C	1.2521	0.2152	0.8923	0.156*	
N1	−0.4170 (14)	0.54122 (16)	0.0321 (9)	0.061 (2)	
O1	0.1218 (10)	0.51313 (14)	0.2839 (7)	0.0612 (17)	
S1	−0.0169 (4)	0.56399 (5)	0.1715 (3)	0.0572 (7)	
Br1	−0.7883 (2)	0.67797 (3)	−0.20868 (16)	0.0951 (6)	

### Atomic displacement parameters (Å^2^)

**Table:**

	*U*^11^	*U*^22^	*U*^33^	*U*^12^	*U*^13^	*U*^23^
C1	0.037 (5)	0.061 (7)	0.033 (5)	−0.002 (5)	0.000 (4)	0.001 (4)
C2	0.061 (6)	0.053 (7)	0.055 (6)	−0.012 (5)	0.014 (5)	0.007 (5)
C3	0.048 (6)	0.071 (8)	0.071 (7)	0.001 (6)	0.001 (5)	−0.002 (6)
C4	0.067 (7)	0.063 (7)	0.048 (6)	0.013 (6)	0.017 (5)	0.005 (5)
C5	0.071 (7)	0.047 (7)	0.083 (7)	0.002 (5)	0.011 (6)	−0.007 (5)
C6	0.046 (5)	0.048 (6)	0.072 (7)	0.002 (5)	−0.002 (5)	0.005 (5)
C7	0.037 (5)	0.074 (7)	0.034 (5)	0.007 (5)	−0.006 (4)	0.012 (5)
C8	0.045 (5)	0.047 (6)	0.054 (6)	−0.001 (5)	−0.017 (5)	0.002 (5)
C9	0.059 (6)	0.036 (6)	0.068 (6)	0.000 (5)	−0.016 (5)	0.017 (5)
C10	0.053 (6)	0.045 (6)	0.056 (6)	−0.003 (5)	−0.001 (5)	0.013 (5)
C11	0.059 (6)	0.053 (7)	0.056 (6)	0.013 (5)	0.008 (5)	0.004 (5)
C12	0.043 (5)	0.056 (6)	0.051 (6)	0.006 (5)	0.000 (4)	0.002 (5)
C13	0.049 (6)	0.056 (7)	0.054 (6)	0.001 (5)	0.002 (5)	0.007 (5)
C14	0.031 (5)	0.079 (8)	0.066 (6)	−0.012 (5)	−0.005 (5)	0.003 (5)
C15	0.051 (6)	0.048 (7)	0.071 (6)	0.011 (5)	0.003 (5)	0.009 (5)
C16	0.046 (6)	0.076 (8)	0.054 (6)	0.015 (5)	0.006 (5)	0.006 (5)
C17	0.051 (6)	0.053 (7)	0.082 (7)	0.006 (5)	0.003 (5)	0.013 (5)
C18	0.057 (6)	0.058 (7)	0.081 (7)	0.005 (5)	−0.010 (5)	0.004 (6)
C19	0.061 (6)	0.047 (7)	0.085 (7)	−0.001 (5)	−0.003 (5)	0.003 (5)
C20	0.070 (7)	0.050 (7)	0.090 (8)	−0.005 (5)	0.002 (6)	0.016 (6)
C21	0.074 (8)	0.054 (8)	0.171 (12)	0.015 (6)	−0.017 (8)	0.031 (8)
N1	0.060 (5)	0.037 (5)	0.079 (6)	−0.016 (4)	−0.010 (4)	0.003 (4)
O1	0.050 (4)	0.058 (5)	0.069 (4)	−0.012 (3)	−0.010 (3)	0.009 (3)
S1	0.0479 (13)	0.0455 (15)	0.0700 (16)	−0.0069 (12)	−0.0138 (12)	0.0077 (12)
Br1	0.1110 (11)	0.0733 (9)	0.1017 (10)	0.0372 (7)	0.0199 (7)	0.0262 (7)

### Geometric parameters (Å, °)

**Table:**

C1—C2	1.376 (11)	C12—H12B	0.9700
C1—C6	1.407 (12)	C13—C14	1.481 (12)
C1—C7	1.428 (12)	C13—H13A	0.9700
C2—C3	1.381 (12)	C13—H13B	0.9700
C2—H2	0.9300	C14—C15	1.535 (11)
C3—C4	1.343 (13)	C14—H14A	0.9700
C3—H3	0.9300	C14—H14B	0.9700
C4—C5	1.381 (13)	C15—C16	1.497 (12)
C4—Br1	1.895 (9)	C15—H15A	0.9700
C5—C6	1.383 (12)	C15—H15B	0.9700
C5—H5	0.9300	C16—C17	1.510 (11)
C6—H6	0.9300	C16—H16A	0.9700
C7—N1	1.317 (11)	C16—H16B	0.9700
C7—S1	1.746 (8)	C17—C18	1.490 (12)
C8—C9	1.332 (11)	C17—H17A	0.9700
C8—O1	1.339 (9)	C17—H17B	0.9700
C8—S1	1.725 (9)	C18—C19	1.503 (11)
C9—N1	1.367 (10)	C18—H18A	0.9700
C9—H9	0.9300	C18—H18B	0.9700
C10—O1	1.423 (10)	C19—C20	1.525 (12)
C10—C11	1.517 (11)	C19—H19A	0.9700
C10—H10A	0.9700	C19—H19B	0.9700
C10—H10B	0.9700	C20—C21	1.492 (12)
C11—C12	1.494 (12)	C20—H20A	0.9700
C11—H11A	0.9700	C20—H20B	0.9700
C11—H11B	0.9700	C21—H21A	0.9600
C12—C13	1.544 (11)	C21—H21B	0.9600
C12—H12A	0.9700	C21—H21C	0.9600
			
C2—C1—C6	117.2 (8)	C13—C14—C15	114.3 (7)
C2—C1—C7	121.1 (8)	C13—C14—H14A	108.7
C6—C1—C7	121.6 (8)	C15—C14—H14A	108.7
C1—C2—C3	121.2 (9)	C13—C14—H14B	108.7
C1—C2—H2	119.4	C15—C14—H14B	108.7
C3—C2—H2	119.4	H14A—C14—H14B	107.6
C4—C3—C2	121.1 (9)	C16—C15—C14	117.0 (8)
C4—C3—H3	119.4	C16—C15—H15A	108.1
C2—C3—H3	119.4	C14—C15—H15A	108.1
C3—C4—C5	119.8 (9)	C16—C15—H15B	108.1
C3—C4—Br1	121.6 (8)	C14—C15—H15B	108.1
C5—C4—Br1	118.6 (8)	H15A—C15—H15B	107.3
C4—C5—C6	119.8 (9)	C15—C16—C17	114.1 (8)
C4—C5—H5	120.1	C15—C16—H16A	108.7
C6—C5—H5	120.1	C17—C16—H16A	108.7
C5—C6—C1	120.7 (8)	C15—C16—H16B	108.7
C5—C6—H6	119.6	C17—C16—H16B	108.7
C1—C6—H6	119.6	H16A—C16—H16B	107.6
N1—C7—C1	124.7 (7)	C18—C17—C16	115.7 (8)
N1—C7—S1	111.8 (7)	C18—C17—H17A	108.4
C1—C7—S1	123.6 (7)	C16—C17—H17A	108.4
C9—C8—O1	131.4 (9)	C18—C17—H17B	108.4
C9—C8—S1	110.7 (7)	C16—C17—H17B	108.4
O1—C8—S1	117.9 (6)	H17A—C17—H17B	107.4
C8—C9—N1	115.0 (8)	C17—C18—C19	113.5 (8)
C8—C9—H9	122.5	C17—C18—H18A	108.9
N1—C9—H9	122.5	C19—C18—H18A	108.9
O1—C10—C11	110.1 (7)	C17—C18—H18B	108.9
O1—C10—H10A	109.7	C19—C18—H18B	108.9
C11—C10—H10A	109.7	H18A—C18—H18B	107.7
O1—C10—H10B	109.6	C18—C19—C20	114.9 (8)
C11—C10—H10B	109.7	C18—C19—H19A	108.5
H10A—C10—H10B	108.2	C20—C19—H19A	108.5
C12—C11—C10	110.8 (7)	C18—C19—H19B	108.5
C12—C11—H11A	109.5	C20—C19—H19B	108.5
C10—C11—H11A	109.5	H19A—C19—H19B	107.5
C12—C11—H11B	109.5	C21—C20—C19	114.7 (8)
C10—C11—H11B	109.5	C21—C20—H20A	108.6
H11A—C11—H11B	108.1	C19—C20—H20A	108.6
C11—C12—C13	113.6 (7)	C21—C20—H20B	108.6
C11—C12—H12A	108.9	C19—C20—H20B	108.6
C13—C12—H12A	108.9	H20A—C20—H20B	107.6
C11—C12—H12B	108.9	C20—C21—H21A	109.5
C13—C12—H12B	108.9	C20—C21—H21B	109.5
H12A—C12—H12B	107.7	H21A—C21—H21B	109.5
C14—C13—C12	114.9 (7)	C20—C21—H21C	109.5
C14—C13—H13A	108.5	H21A—C21—H21C	109.5
C12—C13—H13A	108.5	H21B—C21—H21C	109.5
C14—C13—H13B	108.5	C7—N1—C9	113.0 (7)
C12—C13—H13B	108.5	C8—O1—C10	114.0 (6)
H13A—C13—H13B	107.5	C8—S1—C7	89.5 (4)
			
C6—C1—C2—C3	0.6 (13)	C11—C12—C13—C14	−179.7 (8)
C7—C1—C2—C3	177.3 (8)	C12—C13—C14—C15	177.0 (7)
C1—C2—C3—C4	−0.6 (14)	C13—C14—C15—C16	−177.5 (8)
C2—C3—C4—C5	0.9 (14)	C14—C15—C16—C17	174.3 (8)
C2—C3—C4—Br1	−177.2 (7)	C15—C16—C17—C18	−178.7 (9)
C3—C4—C5—C6	−1.3 (14)	C16—C17—C18—C19	178.0 (8)
Br1—C4—C5—C6	176.9 (7)	C17—C18—C19—C20	−177.2 (9)
C4—C5—C6—C1	1.3 (14)	C18—C19—C20—C21	176.3 (9)
C2—C1—C6—C5	−0.9 (13)	C1—C7—N1—C9	−178.2 (8)
C7—C1—C6—C5	−177.6 (8)	S1—C7—N1—C9	1.8 (10)
C2—C1—C7—N1	−1.1 (13)	C8—C9—N1—C7	−2.0 (12)
C6—C1—C7—N1	175.5 (8)	C9—C8—O1—C10	1.0 (13)
C2—C1—C7—S1	178.9 (6)	S1—C8—O1—C10	179.1 (6)
C6—C1—C7—S1	−4.5 (12)	C11—C10—O1—C8	177.7 (7)
O1—C8—C9—N1	179.5 (9)	C9—C8—S1—C7	−0.2 (7)
S1—C8—C9—N1	1.2 (11)	O1—C8—S1—C7	−178.7 (7)
O1—C10—C11—C12	−179.2 (7)	N1—C7—S1—C8	−0.9 (7)
C10—C11—C12—C13	−178.7 (7)	C1—C7—S1—C8	179.0 (7)
